# Knowledge, attitude and practice of Ethiopian pediatricians concerning childhood eye diseases

**DOI:** 10.1186/s12886-021-01842-5

**Published:** 2021-02-17

**Authors:** Tolosa Tufa Regassa, Kumale Tolesa Daba, Ido Didi Fabian, Aemero Abateneh Mengasha

**Affiliations:** 1 Jimma University, Jimma, Ethiopia; 2 Goldschleger Eye Institute, Sheba Medical Center, Tel Hashomer, Tel-Aviv University, Tel-Aviv, Israel and the International Centre for Eye Health, London School of Hygiene and Tropical Medicine, London, UK

**Keywords:** Knowledge, Attitude, Practice, Childhood blindness, Pediatrician, Jimma, Ethiopia

## Abstract

**Background:**

Eye examination and vision assessment are vital for the detection of conditions that result in blindness. Childhood blindness seriously impacts the development, education, and future employment opportunities of affected children. Pediatricians’ knowledge of eye diseases is critical for the prevention of blindness through early diagnosis, allowing proper treatment and identification of conditions requiring referral to an ophthalmologist to preserve or restore vision. This study aimed to assess the knowledge, attitude, and practice of Ethiopian pediatricians concerning childhood eye diseases.

**Methods:**

We carried out a cross-sectional descriptive study of pediatricians working in various hospitals and clinics in Ethiopia. Participants were selected via a convenient sampling technique. Data were collected using both closed and open-ended semi-structured questionnaires. Responses were entered into EpiData 3.1 and transferred to SPSS version 21.0 software for analysis.

**Results:**

A total of 79 pediatricians participated in the study. Our findings showed that the attitude of all but 2 participants towards improving the management of childhood eye diseases was positive, even though this was not reflected in actual knowledge or practice. Even though attitudes were positive, knowledge was often poor and practice inadequate owing to barriers such as inadequate undergraduate training, lack of ophthalmology options during pediatric residency, and unavailability of ophthalmic equipment.

**Conclusions:**

Participants’ attitudes towards improving treatment for childhood eye diseases are positive, but their insufficient knowledge of eye diseases makes their practice poor in this respect.

**Supplementary Information:**

The online version contains supplementary material available at 10.1186/s12886-021-01842-5.

## Background

Vision is critical for children’s early development and is likely to provide a unifying mechanism through which information perceived through other sensory modalities can be organized and become interrelated [1]. Early detection of visual impairment and providing correct and timely treatment is therefore imperative for the child’s growth and development.

The global estimate of childhood blindness in 2015 was 1.14 million [2]. Causes of blindness in children vary widely between regions, reflecting socio-economic development, cultural practices, coverage of preventive measures (e.g. measles immunization), and access to appropriate eye care and optical services [3, 4]. Corneal scarring due to vitamin A deficiency, measles infection, ophthalmia neonatorum, and the harmful effects of traditional eye remedies are the most common causes of visual impairment in many low-income countries, whereas in high-income countries the main causes are cortical visual impairment, retinal disorders including retinopathy of prematurity (ROP) and disorders of the optic nerve. ROP is a major cause of visual impairment in children in middle-income countries and in urban centers of low-income countries [4–8].

A blind child is more likely to be living under conditions of socioeconomic deprivation, to be more frequently hospitalized during childhood, and to die in childhood than a child not living with blindness. About two-thirds of blind children live in developing countries, and up to 60% of these children who become blind because of keratomalacia are likely to die within 1 year of becoming blind [9, 10].

In Ethiopia, eye problems are among the major public health challenges, with huge economic and social impacts on affected individuals and the society, and the nation at large. Childhood blindness accounts for over 6% of the total blindness burden in this country. Sight-threatening or life-threatening ocular disorders such as congenital cataracts, corneal blindness (mainly as a result of measles and vitamin A deficiency), congenital eye anomalies, retinoblastoma, and glaucoma are common ocular morbidities in Ethiopia [11, 12]. Many of these could be either prevented or treated by early diagnosis and treatment. Pediatricians can play an important part in preventing blindness in children through routine screening of vision at well-child visits. However, early detection and appropriate referral to an ophthalmologist are largely dependent on the pediatrician’s knowledge, attitude, and practice (KAP).

In a questionnaire-based study carried out in the USA in 2012 to assess practices, attitudes, and perceived barriers among pediatricians to pediatric vision screening, Couser et al. [13] reported that in Atlanta, GA, USA, the majority of respondents (67%) indicated that they did not begin formal visual acuity (VA) testing until children were at least 3 years old. The most frequently reported barriers to screening were inadequate training (48%) and time required for examination (42%) [13]. In a survey carried out in 1995 in Illinois, USA, to determine state-wide compliance with the requirement of vision screening by pediatricians, Marcinak et al. [13] reported that 60% of pediatricians tested the VA of children aged 5 years and older, and that half of these pediatricians also tested children aged 2 to 4. The commonest reasons given by those pediatricians for not testing VA were inadequate time (42%), children too young (18%), or that screening would be done at school (18%) [14]. In a national sample of pediatricians surveyed to evaluate preschool vision screening practices, Kemper and Clark [15] found that the rate of VA screening in 3-year-old children was low (35%) but was increased in children aged 4 (73%) and 5 (66%). Commonly reported barriers to vision screening were that screening was too time-consuming (49%) and children were uncooperative (23%). A few pediatricians (3%) in that survey affirmed that screening was unnecessary because vision problems would be identified elsewhere (e.g., by the family) [14]. In a study by Wanyama et al. of the KAP of eye diseases in children among pediatricians in Kenya [16], knowledge was poor in 69.6% of the 125 study respondents. Of the 69.6% of participants in that study who reported carrying out eye examinations in children, only 43.5% did so as a routine part of every child’s examination. Among the 30.4% who reported not doing eye examination, the reasons given were insufficient time to do the examination (39.5%) and not knowing how to do it (31.6%) [15].

In Ethiopia, the distribution of ophthalmologists and eye-care workers in the various regions of the country is currently inadequate compared to that of pediatricians. Despite the prominent role of pediatricians in the prevention of childhood blindness, their KAPs concerning childhood eye diseases are not known. The present study was therefore undertaken to assess the KAPs of Ethiopian pediatricians concerning childhood ocular morbidities.

## Methods

The study population of this cross-sectional descriptive study comprised 79 pediatricians who work in various hospitals and clinics in Ethiopia. Following ethical clearance obtained from the Ethical Review Committee of Jimma University, participants were recruited for the study by convenience sampling during the annual conference of the Ethiopian Pediatric Society held in February 2019. After being informed about the study, the questionnaire developed for this study (provided as Additional file 1), each participant was required to sign a written consent form before completing a closed- and open-ended self-administered structured questionnaire. These were distributed on tables in the conference room and were later collected by two well-oriented ophthalmic nurses and one optometrist. Only those pediatricians who had signed their informed consent were included in the study population. The collected data were checked for completeness and were then coded, entered into EpiData version 3.1, and exported to SPSS version 21. Descriptive statistics were expressed as frequency, percentage, mean, range, and standard deviation. The overall KAP of each participant was analyzed in detail and presented in tabular form. A Chi-square test was used to test the associations between different variables, and a *P*-value of < 0.05 was interpreted as statistically significant.

Participants’ knowledge and practice were categorized according to Bloom’s cut-off points into ‘good’ (> 80%), ‘moderate’ (60–80%), or ‘poor’ (< 60%) [16]. Their responses in the part of the questionnaire assessing attitudes to childhood eye diseases were categorized as positive or negative statements on the Likert scale. Scores in that section varied from 11 to 55, and all individual answers by each participant were summed to obtain his or her total score, which was then classified into 1 of 3 attitude levels: ‘positive’ (scores of 44 to 55 were equivalent to 80%–100%); ‘neutral’ (scores of 33 to 43, equivalent to 60% − 80), and ‘negative’ (scores < 33, equivalent to less than 60%).

The following operational definitions were used: *Children:* age < 16 (UNICEF); *Knowledge:* what eye diseases the pediatrician is familiar with in children; *Attitude:* how pediatricians feel and what they believe concerning eye disease in children; *Practice:* actions the pediatrician intends to take to prevent blindness in children.

## Results

### Background characteristics of participants

Questionnaires were completed and returned by 79 of the 123 eligible pediatricians who were attending the conference, a response rate of 64.2%. The respondents comprised 45 males and 34 females. Their mean age was 37.8 years (SD = 9.8 years), range 26–66 years, and their mean duration of medical practice was 8.2 years, range 1–33 years (Table 1).

**Table 1:** Background characteristics of participants (*n* = 79)

Characteristics	Variables	n (%)
Sex	Female	34 (43.0)
	Male	45 (57.0)
Age (years)	21–30	27 (34.2)
	31–40	24 (30.4)
	41–50	26 (32.9)
	51–60	2 (2.5)
Duration of practice (years)	1–10	54 (68.4)
	11–20	20 (25.3)
	21–30	1 (1.3)
	> 30	> 4 (5.1)
Type of practice	Government	57 (72.2)
	Private	17 (21.5)
	Non-governmental organization (NGO)	5 (6.3)
Place of practice	Specialist hospital	56 (70.9)
	General hospital	20 (25.3)
	Primary hospital	3 (3.8)
Duration of undergraduate training in ophthalmology ^a^(weeks)	2	25 (31.6)
	3	15 (19.0)
	4	17 (21.5)
	5	8 (10.0)
	6	12 (15.2)
	Not sure	2 (2.5)
Duration of postgraduate training in ophthalmology (weeks)	2	2 (2.5)

^a^Undergraduate ophthalmology training at a medical school in Ethiopia

### Knowledge of childhood eye diseases

Knowledge of the 79 pediatricians about childhood diseases, as reflected by their responses on the questionnaire, is indicated in Table 2. As recorded in the table, the large majority 63; /79.3%) of respondents (Table 2) did not know signs of poor vision in children, and the rest had only poor knowledge of such signs, with ‘poor school performance’ being the one most frequently recognized. Only a few knew the definition of blindness according to the World Health Organization (WHO). There was good overall awareness of more than one ocular sign of vitamin A deficiency, the most frequently recognized signs being Bitot’s spots, xerosis, and keratomalacia.

**Table 2:** Knowledge of pediatricians about childhood eye diseases

Characteristic	Variable	n (%)
Do you know of any signs of poor vision in children?	No	63 (79.8)
	Yes	16 (20.3)
	Poor school performance	12 (15.2)
	Nystagmus	2 (2.5)
	Frequent eye rubbing or blinking	1 (1.3)
	Head tilted to one side	1 (1.3)
Knowledge of WHO definition of blindness	VA < 3/60	25 (31.6)
	No light perception	20 (25.3**)**
	Don’t know	22 (27.8)
	VA < 6/60, but ≥3/60	12 (15.2)
Knowledge of ocular signs of vitamin A deficiency	Bitot’s spots	66 (83.5)
	Ocular xerosis	63 (79.7)
	Keratomalacia	63 (79.7)
	Night blindness	19 (24.1)
Knowledge of causes of leukocoria in children	Retinoblastoma	76 (96.2)
	Cataract	57 (72.2)
	Retinopathy of prematurity	40 (50.6)
	Retinal detachment	17 (21.5)
When to refer a child with leukocoria?	Immediately	79 (100)
Is refractive error correctable?	Yes	76 (96.2)
	Don’t know	3 (3.8)
Ways of refractive error correction	Spectacles	65 (82.3)
	Surgery	39 (49.4)
	Contact lens	38 (48.1)
Systemic illnesses in children associated with congenital cataracts	TORCH syndrome	74 (93.7)
	Metabolic disorder	20 (25.3)
	Don’t know	2 (2.5)
Do you know about retinopathy of prematurity?	Yes	77 (97.5)
	No	2 (2.5)
What is the age to screen for retinopathy of prematurity?	4–6 weeks	67 (84.8)
	Don’t know	7 (8.9)
	6–12 months	5 (6.3)
Knowledge about presentation of congenital glaucoma	*Skipped the question*	55 (69.6)
	Buphthalmos(enlarged eye)	14 (17.7)
	Lacrimation	13 (16.5)
	Fear of light	5 (6.3)
Knowledge about complication of squint in children	Loss of depth perception	76 (96.2)
	Lazy eye	72 (91.1)
	Social stigma	46 (58.2)
Knowledge of causes of tearing in infant	Conjunctivitis	55 **(**69.6)
	Nasolacrimal duct obstruction	46 (58.3)
	Congenital glaucoma	27 (34.2)
	*Skipped the question*	6 (7.6)
	Foreign body	1 (1.3)
Knowledge about presenting signs of retinoblastoma	Leukocoria	56 (70.9)
	Proptosis	45 (57.0)
	*Skipped the question*	8 (10.1)
	Squint	3 (3.8)
Treatment of retinoblastoma	Surgery	74 (93.7)
	Chemotherapy	70 (88.6)
	Radiation therapy	28 (35.4)
	Not treatable	2 (2.5)

*VA* visual acuity

As also shown in Table 2, all but 3 of the 79 pediatricians knew that refractive error is correctable. Most of those responded that it can be corrected by spectacles, with smaller numbers indicating that it can be corrected by the use of contact lenses or by surgery. There was a good knowledge of TORCH infection in children as a cause of cataract-associated systemic illness, but knowledge of the effects of other metabolic disorders was poor. All but 2 respondents knew about ROP, and most of them correctly said that the time to screen infants for ROP was at 4–6 weeks. Knowledge about the presenting features of congenital glaucoma was moderate to poor, as was recognition of conjunctivitis and/or nasolacrimal duct obstruction as causing tearing in infants.

Table 2 shows that in response to the question about presenting signs of retinoblastoma, more than half mentioned leukocoria, and a somewhat smaller number mentioned proptosis. For the treatment of retinoblastoma, 74 of the 79 of the respondents listed surgery and 70 listed chemotherapy.

All in all, the knowledge scores of the majority of respondents as shown in Table 2 were below 60%, indicating that the overall level of knowledge of this study population was poor.

### Treatment practice and referral of eye disease cases

Practices of the 79 pediatricians regarding treatment and referral of eye diseases in children are recorded in Table 3. To summarize those results, 68 responded that they do perform such eye examinations, but only 29 reported performing them routinely. More than half (59/79) perform a pupillary examination, and among those who do not, the most frequently cited reason was inadequate training. For children with red eyes, 5 reported an immediate referral for eye care. All the rest said that they prescribe eye drops; among these 74 responders, almost half said that failing improvement they refer the child for eye care, while a small number (6/74) said that following eye-drop administration they refer the child immediately. In the case of squint, the response of the large majority of responders was an immediate referral, while 5 said that they refer if the squint fails to resolve after follow-up. In the case of suspected retinoblastoma, most respondents listed immediate referral, while the remaining 20 reported ordering an imaging examination. For children with congenital cataracts or suspected congenital glaucoma, referral to eye-care workers was the response of nearly all of the pediatricians.

**Table 3:** Practices of treatment and referral of various eye disease cases in children

Question	Respondent’s answer(s)	n (%)
Do you do eye examinations on children? (*n =* 79)	Yes	68 (86.1)
	No	11 (13.9)
How frequently do you perform eye examinations? (*n* = 68)	Every visit	29 (36.7)
	When I see an eye problem	21 (26.6)
	When caregiver complains	18 (22.8)
Which test do you usually do? (*n =* 68)	Pupillary response	59 (74.7)
	Ocular motility	39 (49.4)
	Visual acuity	31 (39.2)
	Fundus examination	1 (1.3)
What is/are reasons not to do eye examination? (*n* = 11)	Not adequately trained	5 (6.3)
	Not my responsibility	1 (1.3)
	Time-consuming	1 (1.3)
	Difficult (i.e. children uncooperative)	2 (2.5)
	I do not know how to examine	2 (2.5)
How do you manage children with red-eye (*n =* 79)	Give eye drops and refer if no improvement	39 (49.4)
	Give eye drops	29 (36.7)
	Give eye drops and refer immediately	6 (7.6)
	Refer immediately	5 (6.3)
What do you do for a child who squints? (*n =* 79)	Refer immediately to eye-care worker	74 (93.7)
	Follow up and refer if it fails to resolve	5 (6.3)
What do you do for a child in whom you suspect retinoblastoma? (*n =* 79)	Immediately refer to eye-care center	59 (74.7)
	Order CT-scan	17 (21.5)
	Order B-scan ultrasonography	3 (3.8)
What do you do for a child with a congenital cataract? (*n* = 79)	Immediately refer to eye-care worker	79 (100.0)
What do you do for a child you might suspect of having congenital glaucoma? (*n =* 79)	Refer to eye-care worker immediately	77 (97.5)
	Give eye drops and follow up	2 (2.5)
What do you do for a child with congenital tearing? (*n* = 79)	Immediately refer to eye-care worker	47 (59.5)
	Maintain observation	23 (29.1)
	Reassure the family	6 (7.6)
	Give eye drops and send home	3 (3.8)

### Attitude of participants

Table 4 shows that nearly all of the 79 responding pediatricians had a positive attitude to providing care in children with eye diseases.

**Table 4:** category of attitude on the Likert Scale (*n* = 79)

Category	n (%)
Positive attitude	75 (94.9%
Neutral attitude	4 (5/1%)
Total	79

## Discussion

From the responses of the pediatricians who completed the questionnaire on which this study was based, it is evident from Table 2 that the large majority (63/79) had poor knowledge of the signs of poor vision in children—although poor school performance, possibly a late sign of poor vision, was mentioned by an additional 12 responders. Those findings may be explained both by insufficient course time devoted to undergraduate ophthalmology, as the majority of participants had taken it for only 2 weeks and by the absence of an ophthalmology attachment during residency. The table also shows that a large majority did not know the WHO definition of blindness. To the best of our knowledge, the literature contains very few studies that could serve for comparison in this respect. Most of our respondents mentioned more than one ocular sign of vitamin A deficiency (Bitot’s spots, xerosis, and keratomalacia), reflecting a moderate to a good level of knowledge that might be explained by the fact that vitamin A deficiency is a common problem in pediatric practice and is likely to be included in their pediatric training curriculum because vitamin A deficiency in children is a public health problem in Ethiopia.

Concerning leukocoria, all but 3 respondents knew that retinoblastoma is a possible cause, but knowledge of cataract as a cause was only moderate and knowledge of ROP as a cause of leukocoria was poor (Table 2). These findings are comparable with those reported in the study carried out in Kenya, in which retinoblastoma was mentioned by 90.60% and cataract **by** 74.36% of the respondents as causes of leukocoria [15]. Regarding knowledge of systemic illnesses associated with congenital cataract, we found that such knowledge ranged from good to poor (Table 2): nearly all participants mentioned TORCH infection, while only about a quarter mentioned metabolic disorder, similar to the findings in the Kenya study (94.11 and 28.75%) respectively [16].

Poor knowledge about the presentation of congenital glaucoma was indicated by the fact that as many as 55 respondents skipped the question (Table 2). Notably, buphthalmos, which was included among the questions in this group, was listed by only 17.7% of respondents, compared to 36.89% in the Kenya study [15]. The discrepancy can be attributed to the likelihood that this sign is only rarely recognized by parents as a reason to seek health care for their child, as the big eye is considered**,** in Ethiopian society, to be a beautiful feature. Knowledge of the various causes of tearing in infants was poor, as the mean score of our respondents was only 40.9%. We are not aware of studies that can be cited for comparison.

About the presenting signs of retinoblastoma, Table 2 also shows that knowledge of leukocoria as a presenting symptom was moderate (listed by 70.9% of our respondents compared to 86.07% in the Kenya study), while knowledge of proptosis as a presenting symptom was poor (57% compared to 54.10% in Kenya). The large majority of our respondents listed surgery and chemotherapy as treatment modalities for retinoblastoma (Table 2), indicating a good level of knowledge compared with the Kenya study where 94.40% of respondents knew that retinoblastoma is a treatable disease [16].

Findings to the clinical practice of Ethiopian pediatricians in dealing with childhood ocular illness (Table 3) were good in that most of the participants reported performing eye examination in children, but were also poor, in that only a few of them do this as a routine part of every child’s visit. The practice was moderate for pupillary response testing (which in our study was the test most frequently performed, whereas other authors have reported that the test they most commonly performed in children was VA [14, 16]; this difference might be attributable to the unavailability of a VA chart in most pediatric departments. Inadequate training was the reason most commonly cited in our study by the few who reported that they do not perform an eye examination. Regarding cases of suspicious retinoblastoma, a majority of respondents (about three quarters) said that they immediately refer the child to an ophthalmologist (reflecting a moderate level of practice), while the rest listed their practice of ordering CT-scan and/or ultrasonography. Further study is needed to identify the characteristics suggestive of retinoblastoma on those imaging modalities.

Overall, the attitude of nearly all of the pediatricians in this study towards the management of childhood eye diseases was positive (Table 4) compared to the study in Kenya [15]. Logistic regression analysis of the association showed no statistically significant association between socio-demographic factors and pediatricians’ attitudes to childhood eye morbidities.

## Conclusion

The results of this study demonstrated that even though the participating pediatricians, in general, were found to have moderate or (mainly) poor knowledge and low levels of practice of childhood eye diseases, they exhibited high levels of interest in improving both their knowledge and their practice in aspects such as diagnosis and management of ophthalmic problems and the usage of ophthalmic medications and simple diagnostic instruments. These findings thus demonstrate that centers of academic training in pediatrics and child health need to include courses in pediatric ophthalmology in their curricula for residents. Besides, child-health institutions should ensure that important ophthalmic equipment such as VA charts and direct ophthalmoscopes are available to their health-care workers.

### Strength and limitations of the study

This study is the first to evaluate KAP concerning childhood eye diseases encountered by pediatricians in Ethiopia. Its strength is that it addresses the previously neglected issue of finding ways to overcome childhood blindness, an affliction that continues to profoundly challenge the developing world. The limitations of the study stem from the fact that the data obtained were based on both closed-ended questions which might induce high false-positive, and on self-reported information, a method of investigation which, if used exclusively, is likely to lower the accuracy of the results obtained.

## Supplementary Information

**Additional file 1.**

## Data Availability

The datasets used and analyzed during the current study available from the corresponding author on reasonable request.

## Abbreviations

KAP: Knowledge, attitude, and practice
NGO: Non-governmental organization
ROP: Retinopathy of prematurity
TORCH: Toxoplasmosis, rubella, cytomegalovirus, herpes simplex
UNICEF: United Nations Children’s Fund
VA: Visual acuity
WHO: World Health Organization
