# Supplementary material for: Knowledge, attitude and practice of Ethiopian pediatricians concerning childhood eye diseases
Source: BMC Ophthalmol. 2021 Feb 17;21:91. doi: 10.1186/s12886-021-01842-5 (PMC7890964; doi:10.1186/s12886-021-01842-5)
Supplement: Supplementary file 1 — Additional file 1. [file 12886_2021_1842_MOESM1_ESM.docx]

**Appendix: Questionnaires**

**Section 1: Background**

| 1. Age | ------------- years |
| --- | --- |
| 1. Sex | 1. Male 2. Female |
| 1. For how long have you been practicing as a pediatrician? | -------------------- years |
| 1. What is the type of your practice? | 1. Government 2. private practice 3. NGO |
| 1. Where do you practice? | 1. Primary hospital 2. General hospital 3. Specialized hospital |
| 1. For how long did you take an undergraduate ophthalmology course**?** | **--------------------** weeks |
| 1. Did you take any additional courses or training on ophthalmology? | 1. Yes 2. No |
| 1. If yes to question 7, for how long? | **------------------------------------------------------------------------------------------------------------------** |

**Section 2: Knowledge**

| 1. Do you know any sign of poor vision in a child? | 1. Yes 2. No |
| --- | --- |
| 1. If yes to #1, could you list? | 1. ----------------------------- 2. ----------------------------- 3. ------------------------------ |
| 1. When do you say a child is blind? (according to WHO definition) | 1. VA < 6/60, but ≥3/60 2. VA < 3/60 3. If no perception of light (NPL) 4. I don’t know |
| 1. Do you know any ocular signs of vitamin A deficiency? List | 1. **----------------------------------------** 2. **----------------------------------------** 3. **----------------------------------------** |
| 1. What causes of leukocoria (white pupillary reflex) in children do you know? More than one choice is possible. | - 1. Don’t know   2. Retinoblastoma   3. Cataract   4. Retinopathy of prematurity   5. Retinal detachment   6. Others mention **-----------------------------------------------------------------------------------** |
| 1. When should a child with leukocoria be referred to an ophthalmologist? | - 1. Immediately   2. When it causes reduction of vision   3. When the family (caregiver) requests for referral   4. Other (specify) ----------------------------------------------------------------------------------- |
| 1. Is refractive error correctable? | - 1. Yes   2. No   3. Don’t know |
| 1. If yes to question #7, how? More than one choice is possible | - 1. Spectacle   2. Contact lens   3. Surgery   4. Don’t know |
| 1. Do you know of any systemic illnesses in children associated with congenital cataracts? List in specific | - 1. **----------------------------------**   2. **----------------------------------**   3. **----------------------------------**   4. **----------------------------------**   5. **----------------------------------** |
| 1. Do you know the retinopathy of prematurity (ROP)? | - 1. Yes   2. No |
| 1. If yes to question #10, which babies are likely to develop ROP? More than one choice is possible. | - 1. Preterm baby   2. Sick baby requiring oxygen   3. Weight less than 1500gm   4. Don’t know   5. Other, specify--------------------------------------------------- |
| 1. If yes to question #10, when should the first eye examination be performed to detect ROP? | - 1. 4-6 weeks of age   2. 6-12 months of age   3. When the caregiver request   4. Don’t know |
| 1. If you suspect ROP in a newborn what would you do? | List ------------------------------------------------------------------------------------------------------------------------------------------------------- |
| 1. When do you suspect glaucoma in a child? | List -------------------------------------------------------------------------------------------------------------------------------------------------------------- |
| 1. What is a squint/strabismus? | - 1. Don’t know   2. Crossed eye   3. Others, list -------------------------------------------------------------------------------------------------- |
| 1. Do you know any complication of squint? More than one choice is possible | - 1. Lazy eye/amblyopia   2. Loss of depth perception   3. Social stigma   4. Others, specify ----------------------------------------------------------------------------------- |
| 1. Is a squint treatable? | - 1. Yes   2. No   3. Don’t know |
| 1. Do you know any causes of tearing in an infant? | - 1. Yes   2. No |
| 1. If yes to question #18, list | - 1. ----------------------------------   2. ----------------------------------   3. ----------------------------------   4. ---------------------------------- |
| 1. What are the presenting signs of retinoblastoma? List | - 1. ----------------------------------   2. ----------------------------------   3. ---------------------------------- |
| 1. Do you think retinoblastoma is curable? | - 1. Yes   2. No   3. I do not know |
| 1. What are the treatments for retinoblastoma? | - 1. ----------------------------------   2. ----------------------------------   3. ---------------------------------- |

**Section 3: Practice**

| 1. Do you do eye examinations on children? | 1. **Yes, 2. No** |
| --- | --- |
| 1. If yes, how frequently do you perform? | - 1. At every visit   2. When caregiver reports child has an eye problem   3. When I see eye problem   4. Others specify -------------------------------------------------------- |
| 1. Which tests do you do usually? | 1. Visual acuity 2. Pupillary response 3. Fundus examination 4. Motility 5. Other, specify ------------------------------------------------------------------------------------------------------ |
| 1. If no to question no-1, what is/are the reason you face in performing eye examination in children? | - 1. Not adequately trained   2. Not my responsibility   3. Time-consuming   4. Difficult (i.e. children uncooperative)   5. I do not know how to examine   6. Other (specify) ------------------------------------------------------------------------------------ |
| 1. How do you manage children with red eyes? | - 1. Refer immediately to eye-care worker   2. Give eye drops (specify the eye drops often prescribed) ------------------------------   3. Give eye drops and refer immediately to eye-care worker   4. Give eye drops and refer if no improvement   5. Other (specify--------------------------------------------------------- |
| 1. What do you do for a child with squints? | - 1. Refer immediately to eye-care worker   2. Give eye drops (specify the eye drop) ---------------------------------------------   3. Follow up and refer if it doesn’t resolve   4. Other (specify) ----------------------------------------------------------------------------------- |
| 1. What do you do for the child you suspect retinoblastoma? | - 1. Immediately refer to an ophthalmologist   2. Give eye drop and send home   3. Order B-scan ultrasonography   4. Order CT-scan   5. Other (specify) ----------------------------------------------------------------------------------- |
| 1. What do you do for a child with a congenital cataract? | - 1. Immediately refer to an ophthalmologist   2. Observation   3. Give eye drop and send home   4. Give eye drop and follows |
| 1. What do you do with the child you might suspect congenital glaucoma? | - 1. Refer to eye-care worker immediately   2. Observation   3. Give eye drops and follow (specify eye drop) and ---------------------------------------------------------   4. Other (specify--------------------------------------------------------- |
| 1. What do you do for a child with congenital tearing? | - 1. Immediately refer to eye-care worker   2. Give eye drop and send home   3. Observation   4. Reassure the family |

**Section 4: attitude**

**For the next part indicate the level of your agreement with the statement provided**

| \| **Statement** \| \| --- \| | **Strongly agree** | **Agree** | **Neutral** | **Disagree** | **Strongly disagree** |
| --- | --- | --- | --- | --- | --- | --- |
| \| Your training adequately equips you to diagnose, manage, and refer children with eye diseases. \| \| --- \| |  |  |  |  |  |
| \| More attention should be given to improve the level of education of pediatricians on eye diseases, the use of ophthalmic medications, and simple diagnostic instruments such as visual acuity charts and funduscopy. \| \| --- \| |  |  |  |  |  |
| Pediatricians can play a significant role in the prevention of childhood blindness. |  |  |  |  |  |
| \| Eye exams in children should be done only when the caregiver complains. \| \| --- \| |  |  |  |  |  |
| \| Eye exams in children can only be done by an eye care worker \| \| --- \| |  |  |  |  |  |
| Childhood blindness is not a public health problem in Ethiopia |  |  |  |  |  |
| \| Vitamin A deficiency is not a common issue in pediatric practice. \| \| --- \| |  |  |  |  |  |
| \| Routine eye examination by a pediatrician could help in the early detection of retinoblastoma \| \| --- \| \|  \| |  |  |  |  |  |
| \| All preterm and low birth weight admitted to NICU needs screening for ROP. \| \| --- \| |  |  |  |  |  |
| \| Children with cataracts require a thorough systemic evaluation by the pediatrician. \| \| --- \| |  |  |  |  |  |
| \| In children, vision impairment can affect development, school performance, and future employment opportunity \| \| --- \| |  |  |  |  |  |
